# Scraps

**Published:** 1901-03

**Authors:** 


### SCRAPS

PICKED UP BY L. M. G.

---

**Clinical Records** (34).—Chaplain: "My poor fellow, this is the day when you are to perish on the scaffold. Have you any requests to make?" Culprit: "Yes, I wish you'd send for a doctor. This cold of mine seems to be getting worse, and it may run into something serious."

**Medical Philology** (XXXVII).—The *Promptorium* has "Drotyn̄ yn' speche. *Traulo*." Mr. Way's note is: "This term, implying difficulty of speech, or stuttering, has not been met with elsewhere. The Ortus renders '*traulus*, a ratelere,' a word equally unnoticed by Glossarists, which occurs also in Cath. Ang. 'To rátylle, *traulare;* a ratyller, *traulus*.'" In his note on these words in the *Catholicon*, Mr. Herrtage says: "Cooper renders *traulus* by 'one that can scant utter his wordes.' 'Ratler in the throte who aptly doth not pronounce. *Traulus*.' Huloet."

**Fashions Change.**—It is interesting to watch the progress and decay of prejudice against particular professions. Solicitors are gradually outgrowing the hostility which prompted the famous free translation of *Nemo repente fuit turpissimus:* "It takes five years to become an attorney." And so, too, with doctors, concerning which calling one may recall a curious anecdote. While Rome was still a pontifical city, an Italian *impresario* wrote to Gounod, asking him, as the ecclesiastical censorship prohibited the appearance of the Devil on the stage, if he could not transform the character of Mephistopheles, making him "*per esempio un medico*"*!* In contemporary works of fiction I fancy that the villain is most commonly a company promoter, though, indeed, in Mr. Fergus Hume's new novel he is an Episcopal chaplain.—C. L. G., in *The Londoner.*

**Medical Missions.**—Dr. Duncan Main, of Hang-chow, Mid China, where is perhaps the largest Medical Mission in the world, certainly the largest in China, thus describes its work:—

> "Every year there are something like 1,000 in-patients and 30,000 or 40,000 out-patients, who are received in two hospitals, one for men and one for women; there are also an opium-refuge, three small hospitals for lepers, a home for the untainted children of lepers, a convalescent home, and a training college for young men. I and my staff are supposed to do itinerating work, to be local treasurer and secretary for the Church Missionary Society, to look after all the native missionaries, and to give an eye to all foreigners who come to Hang-chow. In addition to all this, we hold something like 3,000 services a year, for the medical missionary is not a doctor only, but a missionary first and a doctor afterwards."

Hang-chow is a city of 750,000 people. It is surrounded by a wall more than fourteen miles round, and has a single street five miles long.—*Awake.*

**Literary Refreshment for Doctors.**—In an address, entitled "After Twenty-five Years," delivered at the opening of the session of the Medical Faculty, McGill University, Montreal, Dr. William Osler said: "While medicine is to be your vocation, or calling, see to it that you have also an avocation—some intellectual pastime which may serve to keep you in touch with the world of art, of science, or of letters. Begin at once the cultivation of some interest other than the purely professional. The difficulty is in a selection and the choice will be different according to your tastes and training. No matter what it is—but have an outside hobby. For the hard-working medical student it is perhaps easiest to keep up an interest in literature. Let each subject in your year's work have a corresponding outside author. When tired of anatomy, refresh your mind with Oliver Wendell Holmes; after a worrying subject in physiology, turn to the great idealists, to Shelley or Keats for consolation; when chemistry distresses your soul, seek peace in the great pacifier, Shakspere; and when the complications of pharmacology are unbearable, ten minutes with Montaigne will lighten the burden. To the writings of one old physician I can urge your closest attention. There have been, and, happily, there are still in our ranks notable illustrations of the intimate relations between medicine and literature, but in the group of literary physicians Sir Thomas Browne stands pre-eminent. The *Religio Medici*, one of the great English classics, should be in the hands—in the hearts too—of every medical student. As I am on the confessional to-day, I may tell you that no book has had so enduring an influence on my life. . . . . It was one of the strong influences which turned my thoughts towards medicine as a profession, and my most treasured copy—the second I ever bought—has been a constant companion for thirty-one years,—*comes viæ vitæque.* Trite, but true, is the comment of Seneca—'If you are fond of books you will escape the *ennui* of life, you will neither sigh for evening disgusted with the occupations of the day—nor will you live dissatisfied with yourself or unprofitable to others.'" —*Montreal Medical Journal.*

**Richard Mead.**—Of all the numerous scholars, men of letters, collectors, antiquarians, and intellectual benefactors, whom we owe to a profession which has fostered the learned Linacre (described by his friend Erasmus as "*vir non exacti tantum sed severi judicii*"); the gentle Caius, that zealot of universities; the genial, the ingenious Arbuthnot; the bibliophilic Askew; Freind, the medical annalist; the Ovidian Garth; Radcliffe, the founder of mighty libraries; and Watson, the last of the race of Grand Seigneurs de la Médecine—Mead stands pre-eminent as patron, collector, the friend of arts and letters, of artists and *littérateurs*, the stimulant and stimulator of men's bodies and minds alike. Dr. Richard Mead was born at Stepney in August, 1673, and graduated "Philosophiæ et Medicinæ Doctor" at Padua in August, 1695. In 1696 he settled down in practice at Stepney. In 1703, when only thirty years of age, he became F.R.S., of which Society he was elected vice-president in 1707. His fellowship was due to the analysis of Bonomo's letter to Redi on the genesis of the itch, and his *Account of Poisons.* In 1707 Oxford confirmed his Paduan diploma of M.D., and the friendship of Radcliffe leading upon the latter's death to Mead's succession to his practice, established his title to be considered the head of his profession. Amongst men of letters Mead's reputation stood high. Warburton describes him as "a man to whom all people that pretend to letters ought to pay their tribute on account of his great eminence in them and patronage of them"; and Theobald acknowledges the assistance received from him in the preparation of his Shakspere.

In the National Portrait Gallery is a large oil-painting by Allan Ramsay, a reproduction of which in mezzo-tint by R. Houston forms the frontispiece to the 1762 quarto edition of Mead's works; it was also engraved by B. Baron, 1749, with Mead's arms at foot.

Mead's library consisted of 100,000 volumes, and realised by auction £5,499 4s. 5d.; his pictures sold for £3,417 11s., prints and drawings for £1,920, coins and medals £1,975 17s. 6d., and antiquities and other curiosities £3,245; making a grand total of £16,057 12s. 11d. The sale of the "Bibliotheca Meadiana" was conducted by Samuel Baker, York Street, Covent Garden, in two portions. The first was offered on Monday, the 18th November, 1754, and the sale lasted till December 19th. The second began on April 7th, 1755, and also lasted twenty-eight days. The pictures, prints, and drawings were sold by Mr. Langford, in the Great Piazza, Covent Garden, in January and March, 1755, occupying together seventeen evenings. The title-page of the Catalogue, as befits the classical owner, is in Latin: The books are *Venales sub Hasta* (recalling Cicero's statement that a spear was erected in the Forum when goods were for sale by auction), with a motto from Euripides, "Γεῦμα τὴν ὠνὴν καλεῖ."—Mr. Albert Forbes Sieveking, in *St. Thomas's Hospital Gazette.*

The Bristol Medical Library has a copy of the quarto edition of Mead's works and a fine impression of Baron's engraving, and also a copy of the caricature by Hogarth which was reproduced in volume five of the *Asclepiad.* Quite recently the library has obtained a copy of the Catalogue of the sale with the prices marked.

